# Loss of p53 Expression in Gastric Epithelial Cells of *Helicobacter pylori*-Infected Jordanian Patients

**DOI:** 10.1155/2022/7779770

**Published:** 2022-03-23

**Authors:** Mohammad A. Abu-Lubad, Ghada F. Helaly, Weliam J. Haddadin, Dua'a A. K. Jarajreh, Amin A. Aqel, Munir A. Al-Zeer

**Affiliations:** ^1^Department of Medical Microbiology and Pathology, Faculty of Medicine, Mutah University, Al-Karak, Jordan; ^2^Department of Microbiology, Medical Research Institute, Alexandria University, Alexandria, Egypt; ^3^Royal Medical Services, Amman, Jordan; ^4^Department of Applied Biochemistry, Institute of Biotechnology, Technical University of Berlin, Berlin, Germany; ^5^Department of Molecular Biology, Max Planck Institute for Infection Biology, Berlin, Germany

## Abstract

**Background:**

Around half of the global population is chronically infected with the stomach bacterium *Helicobacter pylori,* making it one of the most common chronic infections worldwide. *H. pylori* induces the production of reactive oxygen species, DNA damage, and accelerates the degradation of the tumor suppressor protein p53, which may lead to cancer development. In this study, we investigated the relationship between *H. pylori* infection and the expression of p53 in gastric mucosa in a group of patients from Jordan.

**Methods:**

In this retrospective case-control study, the epithelium of gastric glands in subjects chronically infected with *H. pylori* was examined for the expression of p53. Paraffin-embedded gastric biopsy samples from the archives for 50 Jordanian patients diagnosed with chronic *H. pylori* infection and 25 samples free of *H. pylori* infection and any other gastric abnormalities were selected. Samples were analyzed for the presence of *H. pylori* as well as p53 expression levels in the mucosa and submucosa by immunohistochemical analyses and Western blotting.

**Results:**

*H. pylori* was detected in the gastric tissues of infected individuals (*n* = 50); whereas, no *H. pylori* infection was detected in uninfected healthy individuals (*n* = 25) using immunohistochemistry. In contrast to the noninfected samples of gastric mucosa, no nuclear p53 expression was detected in the infected samples using immunohistochemistry. In addition, the levels of p53 in *H. pylori*-positive samples detected by Western blotting were significantly lower than those in the negative individuals.

**Conclusion:**

Our data reveal that p53 protein expression decreased in gastric mucosa of patients infected with *H. pylori*. The loss of this tumor suppressor may play a role in the increased risk for tumor initiation associated with *H. pylori* carriage.

## 1. Introduction

*H. pylori* is a Gram-negative bacterium that chronically colonizes the human stomach, with almost one in two humans infected worldwide [1, 2]. Infection is acquired early in life, and it is the strongest identified risk factor for the development of antral gastritis, peptic ulcer, and gastric cancer [3]. *H. pylori* is classified as a group 1 carcinogen by the International Agency for Research on Cancer (IARC). Infection with *H. pylori* is recognized as a necessary but insufficient cause of gastric carcinoma [1, 4]. This is due to the complex interplay between bacterial virulence factors and host factors that determine the progression and chronicity of infection [4]. The most studied *H. pylori* virulence genes are located in a 40 kb genomic region called cytotoxin-associated gene pathogenicity island (cagPAI) [5]. One of the most distinctive virulence genes of cagPAI is the cytotoxin-associated gene A (*CagA*), which is delivered into epithelial cells by a cagPAI-encoded type IV secretion system after bacterial attachment to host cells. *CagA* is an oncoprotein that can disrupt several essential signaling pathways of the host [5].

The development of gastric cancer is thought to be a multistep and multifactorial process, including activation of oncogenes, inhibition of tumor suppressor genes, oxidative stress, and DNA damage induced by *H. pylori*. The sequence of events starting from gastritis, atrophy, intestinal metaplasia, dysplasia, and finally gastric cancer can take several decades to develop [5].

*H. pylori* infection remains highly prevalent in most developing countries due to socioeconomic status and hygienic conditions [6]. The prevalence has been reported as 79.1%, 63.4%, and 54.7% in Africa, Latin America, and Asia, respectively, while in North America, it is only around 37.1% [2]. In Jordan, the nationwide seroprevalence of *H. pylori* was around 88.6% in 2018 [7]. The prevalence of *H. pylori* in Jordanian patients with gastritis and peptic ulcer, intestinal-type adenocarcinomas, and adenocarcinomas was 82%, 55.6%, and 48.8%, respectively [8, 9]. In 2018, we demonstrated that all dental plaque samples collected from a group of Jordanian volunteers tested positive for *H. pylori* [10].

It has been reported that in gastric metaplasia and adenocarcinoma, *CagA*-positive *H. pylori* strains contribute to a loss of function of p53 [11, 12], while other studies have found p53 to be expressed in the mucosa of patients with preneoplastic lesions infected with *H. pylori* [13, 14]. Moreover, in patients with chronic gastritis and gastric ulcer, expression of wild-type p53 was found to be upregulated in the inflamed tissues [15]. Increased expression of wild-type 53 has also been found in response to *H. pylori* infection in vitro [16, 17]. Another study found that *H. pylori* infection can activate the AKT serine/threonine kinase 1 (Akt1) in gastric epithelial cells. In turn, Akt1 leads to activation of the E3 ligase human double minute 2 (HDM2) and subsequent degradation of p53 [18].

Induction of p53 degradation is considered the most common phenotype related to oxidative stress and DNA damage during infection, which is accompanied by altered cell survival and apoptosis [19]. Eventhough the prevalence of *H. pylori* in Jordan is high, no studies have so far examined the expression levels of p53 in gastric mucosa of infected patients. Therefore, in this study, we analyzed p53 expression in gastric tissues from Jordanian patients infected with *H. pylori*.

## 2. Materials and Methods

### 2.1. Human Stomach Samples

A retrospective case-control study was conducted on formaldehyde-fixed paraffin-embedded (FFPE) gastric antrum biopsies from *H. pylori*-negative (*n* = 25) collected from 15 males and 10 females and *H. pylori-*positive (chronic *H. pylori* pangastritis) (*n* = 50) collected from 35 male and 15 female patients. Samples were collected between 2015 and 2017 from the archives of the pathology departments from two selected referral medical centers in central (King Hussein Medical City) and north (Al-Karak Governmental Hospital) Jordan. Inclusion criteria were age 15–80 years and positive histopathology for *H. pylori* in the gastric glands for the positive samples and absence of *H. pylori* for the control samples. Exclusion criteria were *H. pylori* eradication therapy during the three months before the biopsy as well as a history of gastric surgery or gastric cancer. According to the records, all patients were clinically suffering from abdominal pain, weight loss, epigastric pain, vomiting, and dyspepsia. Gastric samples that were histopathologically diagnosed as having no *H. pylori* infection or any other abnormalities were used as negative controls.

### 2.2. Immunohistochemistry

Formalin-fixed, paraffin-embedded gastric biopsies were subjected to immunohistochemical staining as described before [20]. Five *μ*m block sections were deparaffinized in xylene and rehydrated in a graded series of ethanol. Then, samples were heated at 95°C for 15 min using antigen retrieval buffer for antigen retrieval (Dako, USA). Samples were then incubated with 3% H_2_O_2_ for 30 min at room temperature to block the endogenous peroxidase activity. The samples were blocked with 1% bovine serum albumin in PBS for one hour at room temperature. Samples were then stained for p53, *H. pylori*, and DNA using monoclonal mouse anti-p53 DO-1 antibody (sc-126, Santa Cruz) (1 : 100 dilution in blocking buffer), polyclonal rabbit anti-urease antibody (sc-21016, Santa Cruz) (1 : 100 dilution), E-cadherin (1 : 100, sc8424), and DAPI for DNA staining (Sigma-Aldrich) for two hours at room temperature. Secondary labeled antibodies for immunofluorescence and Western immunoblot analyses were purchased from Jackson ImmunoResearch Laboratories as published before [21].

### 2.3. Western Blotting

Total protein was extracted from the paraffinized tissues according to the manufacturer's instructions (Qproteome FFPE Tissue, Qiagen). Briefly, using xylene, the deparaffinized sections were incubated in 100 *μ*l extraction buffer. After centrifugation, extracted proteins were recovered in the supernatant. Recovered proteins were mixed directly with sample buffers and boiled at 95°C for 10 min. Then, gel electrophoresis was carried out using the vertical BioRad Mini-Protean II electrophoresis system, USA. Equal amounts of protein were subjected to 12% sodium dodecyl sulfate-polyacrylamide gel electrophoresis (SDS-PAGE). Proteins were separated under reducing conditions for two hours at 120 V. Protein bands were transferred electrophoretically onto Immobilon-P polyvinylidene difluoride membranes (Millipore, MA, USA) using a BioRad electroblotting system (BioRad Mini Trans-Blot Electrophoretic Transfer Cell, USA) [22]. Finally, the transfer was carried out for three hours using 250 mA at 4°C. The membranes were blocked with 5% fat-skim milk in Tris-buffered saline (TBS) pH 7.5, containing 0.05% Tween 20 for one hour at room temperature (22°C). Next, membranes were incubated for one hour at room temperature with anti-p53 and anti-*β*-actin antibodies diluted in TBS-0.05% Tween 20. The membranes were washed 3 times for 20 min with TBS-0.05% Tween 20 and then incubated with secondary antibodies conjugated with horseradish peroxidase. Signal detection was performed with the enhanced chemiluminescence system (ECL, Amersham), by mixing chemiluminescence solutions one and two at a 1 : 1 ratio.

## 3. Results

### 3.1. Detection of *H. pylori* in Gastric Biopsies Using Immunohistochemistry

**Figure 1:**
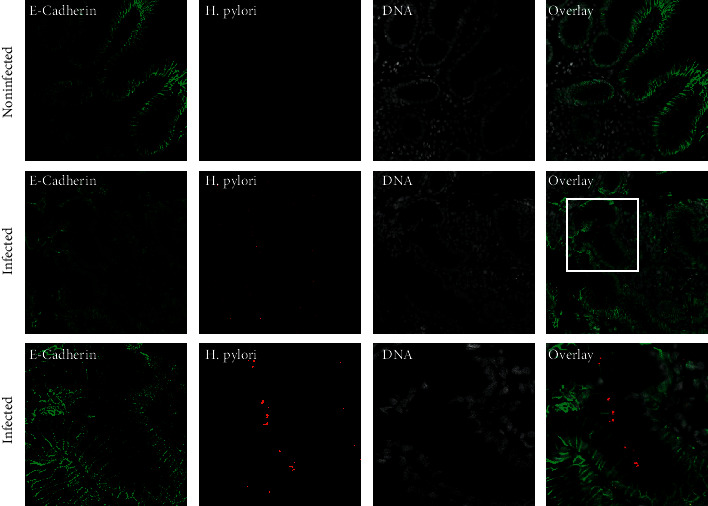
Detection of *H. pylori* in gastric biopsy specimens. Sections of gastric glands obtained from *H. pylori*-positive patients fluorescently labeled for the epithelial marker E-cadherin (green), *H. pylori* (red), and nuclear DNA using DAPI (white). Most of the gastric glands are heavily infected with *H. pylori.*

The presence of *H. pylori* in the gastric biopsies was confirmed using fluorescence immunohistochemistry with a specific antibody against the urease enzyme of *H. pylori*. Seventy-five biopsies were histologically tested for the presence of *H. pylori* infection (Figure 1). The bacterium was detected at the mucosal surface and in the lumen of gastric glands from all patients with *H. pylori* infection (*n* = 50) (Figure 1). No signal for *H. pylori* was detected in all negative control samples (*n* = 25) (data not shown).

### 3.2. The Expression Level of p53 in Gastric Biopsies Infected with *H. pylori*

**Figure 2:**
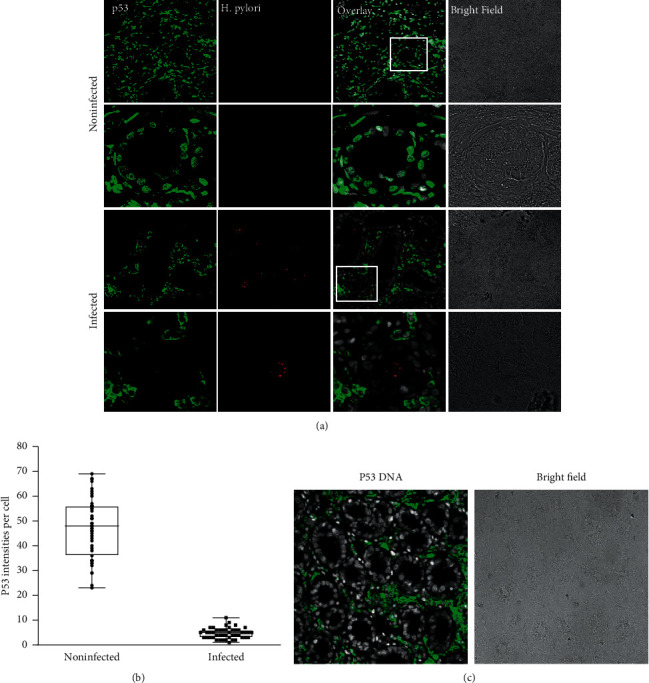
p53 expression in *H. pylori*-infected and noninfected gastric glands. (a) Control gastric glands (no *H. pylori* infection) exhibiting a normal, wild-type pattern of p53 (green) expression in the epithelium and stroma, localized to the nuclei. p53 staining in gastric glands infected with *H. pylori* (red) exhibit a complete loss of nuclear p53 staining in the epithelium, while stromal cells display cytoplasmic staining for p53 (green). (b) Quantification of p53 intensities in gastric tissues revealing a significant decrease in p53 signal in epithelial cells infected with *H. pylori* compared to noninfected cells. Mean pixel intensities of p53 per nucleus were quantified from confocal images with ImageJ. Black bars indicate mean ± SD, ^*∗∗*^*p* < 0.01, as calculated by the *t*-test, *n* ≥ 47. (c) Stromal cells display cytoplasmic staining for p53 (green), while there is no p53 staining in the gastric epithelium.

Fluorescence immunohistochemistry was used to analyze the expression level of p53 protein in *H. pylori*-positive and negative gastric biopsy specimens. In the control samples, the p53 fluorescence signal was strong and detected in most cells irrespective of sectioning sites. Furthermore, the p53 signal was localized to the nucleus of the gastric gland epithelium (Figure 2(a)). In contrast, patients harboring *H. pylori* did not express p53 in the nucleus of gastric gland epithelial cells. Expression levels of p53 in gastric tissue samples were significantly lower compared to control specimens or even undetectable (Figure 2(a)). Quantification of fluorescence signal intensity by ImageJ verified that levels of nuclear p53 protein were significantly decreased in infected gastric glands (Figure 2(b)). Interestingly, in stromal cells of *H. pylori*-infected mucosa, p53 expression was absent from the nucleus and localized in the cytoplasm (Figure 2(c)).

**Figure 3:**
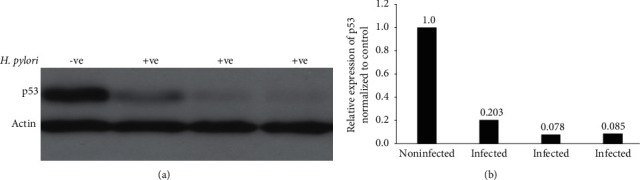
Western blotting analysis of total p53 from paraffinized gastric biopsy specimens. (a) Western blotting of total protein isolated from three *H. pylori*-infected patients and one noninfected patient demonstrating that p53 protein levels are significantly reduced in patients infected with *H. pylori* compared to noninfected patients. Actin was used as a loading control. (b) Band densities from (a) quantified and normalized to corresponding band densities of the *β*-actin loading control.

To further support our findings, we used Western blotting to check and quantify expression levels of p53 from infected vs. uninfected gastric biopsies. In congruence with the immunohistochemical findings, the results from Western blotting showed a significant reduction of p53 expression in gastric biopsies infected with *H. pylori* compared to uninfected biopsies (Figure 3).

## 4. Discussion

*H. pylori* triggers the downregulation and breakdown of p53 protein [12], enabling the bacterium to block apoptosis, one of the main defensive mechanisms in infected cells, and thereby ensure the survival of host cells by breaking down p53 *in vitro* and *in vivo* [23, 24]. The protective function of p53 is also impaired in many forms of cancer [25, 26]. Our present study underpins the direct relationship between chronic infection with *H. pylori* and loss of p53 expression in the epithelium of the gastric mucosa. Loss of nuclear p53 may render the epithelial cells of the stomach vulnerable to the hundreds of mutations that occur every day in almost every cell in our body. Intact p53 protects cells from DNA damage by activating the DNA damage response and subsequently DNA repair or apoptosis [25]. In this manner, cells are normally sheltered against mutations that might predispose them to transformation.

Numerous bacterial infections are now presumed to be a factor in cancer development, but their association is not so decisively proven as for *H. pylori* [6]. *H. pylori'* cagPAI type IV secretion system is recognized as an inflammatory and potentially transforming determinant of *H. pylori* [23]. Our study was not able to determine whether *H. pylori*-positive samples were infected with a *CagA*-carrying strain, and this needs further investigation.

Our data show that in contrast to *H. pylori*-negative glands, gastric epithelial cells infected with *H. pylori* lost nuclear p53 expression. Loss of p53 expression from the epithelium is not only correlated with the presence of *H. pylori* within the lumen of the gastric glands but also to its unique ability to escape the mucosal defense and thereby ensure life-long persistence, which could be related to higher colonization levels and a more pronounced effect on p53 expression.

To date, all the pieces of evidence point towards a role of p53 in tumor suppression as well as its role as a nuclear transcription factor [18]. Therefore, p53 interactions, modifications, and subcellular localization in the cytoplasm might reveal new unexpected aspects of this extensively studied protein and provide a rationale for therapeutic intervention. Interestingly, our findings suggest that p53 is localized to the cytoplasm in stromal cells in patients infected with *H. pylori*. Whether *H. pylori* directly or indirectly influences the subcellular localization and/or posttranslational modification of p53 in the stroma is an open question that will require further investigation. Localization of p53 may affect signal transduction, metabolism, and apoptosis [27]. While several mechanisms by which cytoplasmic p53 leads to the activation of apoptosis have been described to date, and most of the mechanisms regulating its cytoplasmic activities remain largely unknown [28]. Our findings suggest that the possible contribution of cytoplasmic p53 to *H. pylori*-associated oncogenesis and the exact role of the stromal cells in this process represent promising new avenues of investigation.

In conclusion, our results represent an approach that can reveal a causality between a particular bacterial infection and the development of cancer in humans. *H. pylori* induces the loss of p53 from the epithelium, which may enhance cell proliferation and inhibition of apoptosis. Moreover, our data suggest that infected cells may acquire a cancer-related phenotype, thus providing deep insight into the cancer-promoting potential of *H. pylori*. The more conclusive the evidence for the connection between infection and cancer becomes, the more important it is to develop effective vaccines and antibiotics as preventative strategies.

## Data Availability

The data used to support the findings of this study are available from the corresponding author upon request.
